# *Tumor suppressor in lung cancer 1 (TSLC1) *alters tumorigenic growth properties and gene expression

**DOI:** 10.1186/1476-4598-4-28

**Published:** 2005-08-05

**Authors:** Thomas E Sussan, Mathew T Pletcher, Yoshinori Murakami, Roger H Reeves

**Affiliations:** 1 Department of Physiology, Johns Hopkins University School of Medicine, Baltimore, MD 21205-2185, USA; 2 Tumor Suppression & Functional Genomics Project, National Cancer Center Research Institute, Tokyo 104-0045, Japan

**Keywords:** RIS1, Ras-induced senescence, NSCLC, lung cancer, A549, TSLC1

## Abstract

**Background:**

Introduction of cDNA or genomic clones of the tumor suppressor in lung cancer 1 (*TSLC1*) gene into the non-small cell lung cancer line, A549, reverses tumorigenic growth properties of these cells. These results and the observation that *TSLC1 *is down-regulated in a number of tumors suggest that *TSLC1 *functions as a critical switch mediating repression of tumorigenesis.

**Results:**

To investigate this mechanism, we compared growth properties of A549 with the *TSLC1*-containing derivative. We found a G1/S phase transition delay in 12.2. Subtractive hybridization, quantitative PCR, and TranSignal Protein/DNA arrays were used to identify genes whose expression changed when *TSLC1 *was up-regulated. Members of common G1/S phase regulatory pathways such as *TP53*, *MYC*, *RB1 *and *HRAS *were not differentially expressed, indicating that *TSLC1 *may function through an alternative pathway(s). A number of genes involved in cell proliferation and tumorigenesis were differentially expressed, notably genes in the Ras-induced senescence pathway. We examined expression of several of these key genes in human tumors and normal lung tissue, and found similar changes in expression, validating the physiological relevance of the A549 and 12.2 cell lines.

**Conclusion:**

Gene expression and cell cycle differences provide insights into potential downstream pathways of *TSLC1 *that mediate the suppression of tumor properties in A549 cells.

## Background

Non-small cell lung cancer (NSCLC) includes squamous and large cell carcinomas and adenocarcinoma. NSCLC accounts for approximately 75% of all lung cancers diagnosed in the United States [1]. Genetic mutations that activate oncogenes such as *KRAS2 *and *NRAS *[2], and loss of function in tumor suppressors such as *RB1*, *TP53*, *PPP2R1B*, *CDKN2A*, and *TSLC1 *have been demonstrated in NSCLC tumors [3-7].

A549 is derived from an NSCLC adenocarcinoma and displays several properties that are characteristic of transformed cells, including a short cell cycle, loss of contact inhibition, and rapid development of tumors following injection into athymic mice [8]. Introduction of a 1.1 Mb YAC derivative containing the *TSLC1 *gene into A549 restored *TSLC1 *expression to normal levels, creating the stable cell line, 12.2 [8]. 12.2 cells do not develop tumors following injection into athymic mice. TSLC1 protein is down-regulated or lost in NSCLC and a number of other neoplastic diseases, including pancreatic [7], hepatocellular [7], breast [9], prostate [10], nasopharyngeal [11], gastric [12], and cervical cancers [13]. Reduction or loss of *TSLC1 *expression is also observed in cell lines derived from esophageal, ovarian, endometrial, small-cell lung and colorectal tumors [14].

The product of *TSLC1 *is a transmembrane glycoprotein that forms dimers both within a cell and between adjacent cells to promote cell-cell adhesion [15]. This protein contains structural domains homologous to members of the immunoglobulin superfamily, NCAM adhesion proteins, and the nectin family of Ca^2+^-independent cell-cell adhesion proteins [7,16]. It contains two protein-protein interaction domains that are required for tumor suppressor activity [17]. TSLC1 interacts with the actin cytoskeleton through DAL-1, which implies that it plays a role in cell motility [18]. The *TSLC1 *gene has been isolated in a number of different experimental paradigms and has received multiple names as a consequence, including *IGSF4*, *BL2*, *ST17*, *SynCAM1*, *SgIGSF*, *RA175*, and *NECL2 *[16,19-22].

Because *TSLC1 *by itself can reverse tumorigenic and metastatic properties of the highly aggressive A549 cell line, it is of interest to identify downstream effectors of this potent tumor suppressor. Identification of genes or pathways activated by TSLC1 would help to characterize the molecular switch from tumorigenic to non-tumorigenic growth. We characterized the growth differences that result from restoration of *TSLC1 *expression to normal levels and used a number of approaches to identify the underlying changes in gene expression. Several genes involved in Ras-induced senescence, endometrial stromal cell decidualization and trophoblast implantation in the uterus were differentially regulated. Additional genes contributing to cell growth, adhesion, and energy production showed altered expression, as well. We did not find evidence that *TSLC1 *works through any of several previously-characterized cell cycle regulatory pathways. Several expression changes were confirmed in the small amounts of tumor and normal tissue obtained from histological specimens. Thus analysis of this tumor suppressor in the readily accessible A549/12.2 cell system may provide insights into a new gene expression cascade involved in suppression of transformation.

## Results

### TSLC1 Alters Growth Properties of A549 Cells

**Figure 1:**
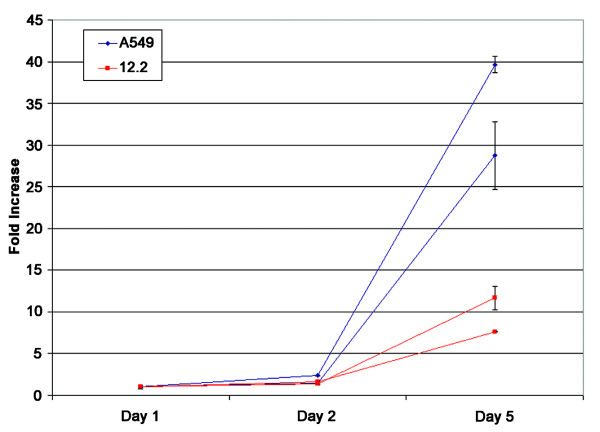
**Cell doubling assay of A549 and 12.2 cells. **Cell number was counted at Day 2 and Day 5, and normalized to Day 1. The results of two independent experiments are shown.

Introduction of the *TSLC1 *gene or cDNA into adenocarcinoma-derived A549 cells restores its expression to normal levels and suppresses many tumorigenic properties of this line [7,8]. We extended observations about the inhibitory effect of *TSLC1 *expression on A549 cell growth [8] by showing that 12.2 cells expanded to only 28% of A549 levels after five days (Table 1 and Fig. 1). This same result was seen with WST-1 reagent, which showed that 48 hours after plating there was a significantly reduced number of viable 12.2 cells relative to A549 (data not shown).

**Table 1:** Expansion rate of A549 and 12.2 cell lines. Expansion rates of A549 and 12.2 cells were determined by counting cells 24 h and 120 h after plating 5 × 10^4 ^cells. Results of two independent experiments are shown.

Cell line	24 hours	120 hours	Fold Increase (120 h/24 h)	Growth Upregulation (A549/12.2)
A549	5.73 × 10^4^	2.27 × 10^6^	39.7	3.40
12.2	4.53 × 10^4^	5.28 × 10^5^	11.7	
A549	2.81 × 10^4^	8.12 × 10^5^	28.9	3.79
12.2	3.59 × 10^4^	2.74 × 10^5^	7.6	

**Figure 2:**
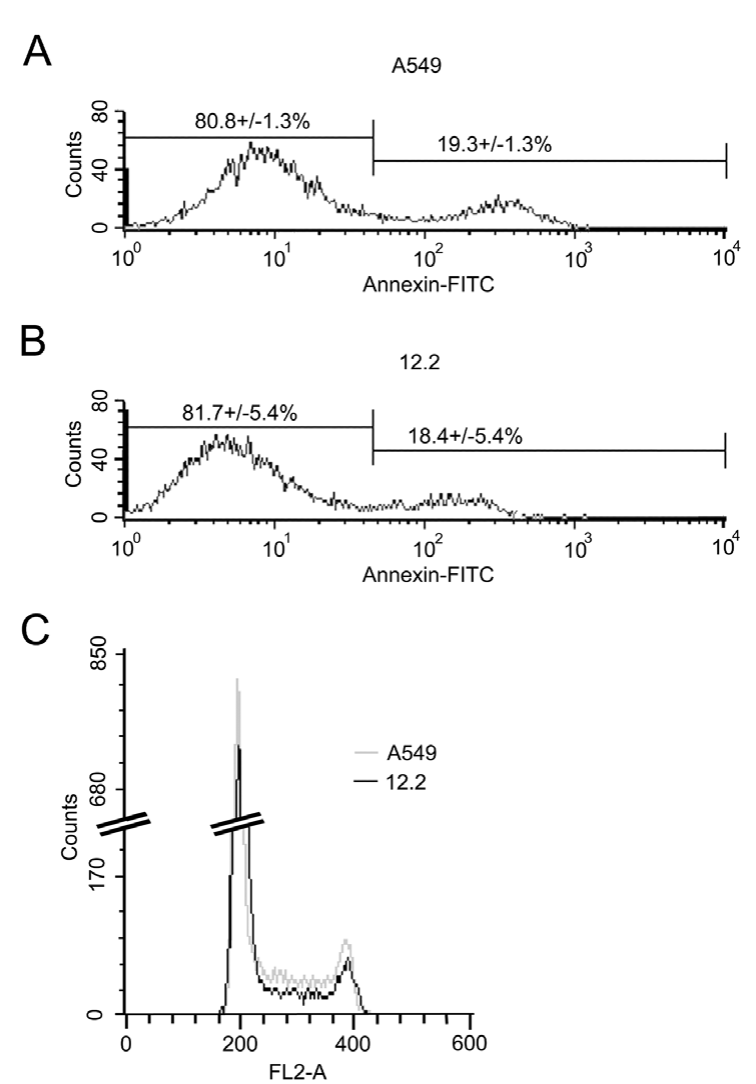
**Flow cytometry analysis of apoptosis and cell cycle in A549 and 12.2**. Histograms of (A) A549 or (B) 12.2 cells stained with annexin V-FITC. Percentages are averages of four experiments; histograms are from one representative experiment. (C) Cell cycle histograms of A549 and 12.2. Cells were fixed, stained with propidium iodide, and analyzed for DNA content by flow cytometry analysis. The left peak represents 2N cells in G1 phase and the right peak represents 4N cells in G2/M phase.

We used flow cytometry to examine how *TSLC1 *affects apoptosis and cell cycle. Rates of apoptosis in A549 and the *TSLC1*-expressing 12.2 cell lines were compared after staining with annexin V. No difference was detected in the number of apoptotic cells (Fig. 2A and 2B). Next, we stained cells with propidium iodide and examined cell cycle profiles of A549 and 12.2 (Fig. 2C and Table 2). The 12.2 cell line showed a significant accumulation of cells in G1 phase (74.4%) compared to A549 (60.4%). Fewer 12.2 cells were seen in S and G2/M phase (17.2 and 8.9%, respectively) when compared to A549 (28.2 and 11.9%, respectively). Thus, the decreased growth rate of 12.2 is due to reduced cell division, which occurs at least in part to a delay at the G1/S phase checkpoint, resulting in delayed progression into S phase.

**Table 2:** Cell cycle profiles of cell lines determined by flow cytometry. Flow cytometry analysis of cell cycle profiles was determined for A549 and 12.2. Percentages were quantified using CellQuest software. A549 and 12.2 profiles are the means of three experiments. The difference between A549 and 12.2 is statistically significant (p < 0.005).

**Cell Cycle Stage**	**Percent Total Cells**	
	
	A549	12.2
G1	60.4+/-0.3	74.4+/-1.8
S	28.2+/-0.5	17.2+/-2.3
G2/M	11.9+/-0.7	8.9+/-1.0

### Expression of Signaling Pathway Genes

Differences in growth rates and cell cycle profiles between A549 and 12.2 led us to examine expression of several known checkpoint and signaling pathway genes using quantitative RT-PCR (qPCR). Alterations in the Ras/p53 pathway result in abnormal G1/S transition in some NSCLC [23]. However, mRNA levels of *HRAS*, *p19*, *RB1*, and *TP53 *were not substantially different between A549 and 12.2 (Table 3). The minor differences in expression levels were not coordinately regulated in a way that would explain lengthening of the G1/S transition in 12.2 cells. We also examined *MYC *and cyclin D1 (*CCND1*), which promote G1 to S phase transition. Neither *CCND1 *nor *MYC*, its upstream regulator, were differentially expressed. Thus, neither of these established pathways appears to be responsible for the G1 delay.

**Table 3:** Comparison of gene expression in A549 and 12.2 cell lines. Genes expressed differentially between A549 and 12.2 cells were identified by qPCR, subtractive hybridization (SH), and/or TransSignal DNA-protein array (TS) as indicated. Relative quantification differences were determined for those genes analyzed by either qPCR or TS. ND, not determined.

**Gene**	**Up-Regulated**	**Assay**	**Fold Expression Difference**	**Functional Class**
Fibrinogen beta chain (*FGB*)	A549	SH/qPCR	2737.5+/-1058.5	Adhesion
Fibrinogen gamma chain (*FGG*)	A549	SH	ND	Adhesion
Adenomatosis polyposis coli (*APC*)*	A549	qPCR	1.7+/-0.2	Cellular Growth
Cyclin D1 (*CCND1*)^†^	A549	qPCR	1.6+/-0.0	Cellular Growth
β-catenin 1 (*CTNNB1*)*	12.2	qPCR	1.1+/-0.0	Cellular Growth
Dishevelled 1 (*DVL1*)*	A549	qPCR	1.1+/-0.3	Cellular Growth
v-Ha-ras Harvey rat sarcoma viral oncogene homolog (*HRAS*)^†^	A549	qPCR	1.4+/-0.3	Cellular Growth
Lymphoid enhancer-binding factor 1 (*LEF1*)*	12.2	qPCR	3.4+/-0.4	Cellular Growth
v-myc myelocytomatosis viral oncogene homolog (*MYC*)^†^	A549	qPCR	1.3+/-0.2	Cellular Growth
Interleukin 23-alpha (*p19*)^†^	A549	qPCR	1.0+/-0.0	Cellular Growth
Tumor protein p53 (*TP53*)^†^	A549	qPCR	1.7+/-0.3	Cellular Growth
Retinoblastoma 1 (*RB1*)^†^	12.2	qPCR	1.3+/-0.4	Cellular Growth
S100 calcium-binding protein P (*S100P*)	A549	SH/qPCR	1680.0+/-955.4	Cellular Growth
Transcription factor 4 (*TCF4*)*	12.2	qPCR	6.8+/-2.0	Cellular Growth
Transcription factor 7-like 2 (*TCF7L2*)*	12.2	qPCR	2.2+/-0.7	Cellular Growth
Transmembrane 4 superfamily member (*TM4SF4*)	A549	SH/qPCR	475.2+/-328.0	Cellular Growth
Centromere protein E (*CENPE*)	A549	SH		Cellular Growth
Heat shock protein 70B (*HSPA6*)	A549	SH/qPCR	2.4+/-0.8	Chaperone
Insulin-like growth factor binding protein 1 (*IGFBP1*)	A549	SH/qPCR	15.9+/-7.4	Decidualization [41]
Retinoid X receptor RXR (DR-1)	A549	TS	2.7	Decidualization [42]
Aldehyde dehydrogenase 1 (*ALDH1*)	A549	SH/qPCR	4.2+/-0.1	Decidualization-Implantation [43]
Annexin A2 (Lipocortin II) (*ANXA2*)	12.2	SH/qPCR	3.1+/-1.0	Decidualization-Implantation [43]
Metallothionein-IF (*MTIF*)	12.2	SH	ND	Decidualization-Implantation [43]
Metallothionein-IG (*MT1G*)	12.2	SH/qPCR	37.1+/-10.4	Decidualization-Implantation [43]
Cadherin 11 (OB-cadherin, osteoblast) (*CDH11*)	A549	SH/qPCR	5.6+/-2.7	Decidualization-Luteal/Adhesion [34,44]
Fibroblast growth factor 9 (*FGF9*)	A549	SH	ND	Decidualization-Proliferation [45]
Promyelocytic leukemia gene (*PML*)	A549	SH	ND	Decidualization-Proliferation
Similar to aldehyde dehydrogenase 6 (*ALDH1A3*)	A549	SH	ND	Dehydrogenase
3-alpha hydroxysteroid dehydrogenase type II (AKR1C3)	A549	SH	ND	Dehydrogenase
Dihydrodiol dehydrogenase 2 (*AKR1C2*)	A549	SH/qPCR	22.9+/-2.4	Dehydrogenase
Kinesin 2, light chain (*KNS2*)	12.2	SH/qPCR	3.9+/-0.0	Intracellular Trafficking
Matrix metalloproteinase 1 (*MMP1*)	12.2	qPCR	1.2+/-0.4	Invasion
Vascular endothelial growth factor (*VEGF*)	12.2	qPCR	4.2+/-0.3	Invasion
Ferritin, heavy chain (*FTH1*)	12.2	SH/qPCR	2.7+/-1.7	Iron Binding
Metallothionein-IE (*MT1E*)	12.2	SH/qPCR	14.8+/-8.5	Metal Homeostasis
Metallothionein-IH (*MT1H*)	12.2	SH	ND	Metal Homeostasis
Metallothionein-IL (*MT1L*)	12.2	SH/qPCR	5.8+/-0.9	Metal Homeostasis
Metallothionein-IR (*MT1R*)	12.2	SH	ND	Metal Homeostasis
Manganese-containing superoxide dismutase (*SOD2*)	A549	SH/qPCR	2.0+/-0.6	Mitochondrial
Microsomal glutathione transferase (*MGST1*)	A549	SH/qPCR	2.3+/-0.1	Mitochondrial
NAD(P)H menadione oxidoreductase 1, dioxin-inducible (NQO1)	A549	SH/qPCR	3.1+/-0.1	Mitochondrial
Solute carrier family 25 member 5 (*SLC25A5*)	A549	SH/qPCR	3.4+/-0.3	Mitochondrial
Myelin proteolipid protein (*PLP*)	A549	SH	ND	Myelin Constituent
Ribosomal protein S6 (*RPS6*)	12.2	SH/qPCR	2.5+/-1.2	Protein Kinase
v-ets erythroblastosis virus E26 oncogene homolog 2 (*ETS2*)	A549	qPCR	1.2+/-0.0	Ras-Induced Senescence [29]
Metallothionein-II (*MTII*)	12.2	SH	ND	Ras-Induced Senescence [29]
Ras induced senescence 1 (*RIS1*)	12.2	SH/qPCR	80.2+/-62.3	Ras-Induced Senescence [29]
Tissue inhibitor of metalloproteinase 1 (*TIMP1*)	12.2	qPCR	2.2+/-0.1	Ras-Induced Senescence [29]
CAAT box general (CBF)	12.2	TS	2.5	Transcription Factor
CCAAT displacement protein (CDP)	12.2	TS	2	Transcription Factor
E2F transcription factor 1 (E2F-1)	12.2	TS	2.4	Transcription Factor
Early growth response (EGR)	12.2	TS	3.4	Transcription Factor
Estrogen receptor (ERE)	12.2	TS	2.5	Transcription Factor
GATA binding protein (GATA)	12.2	TS	2.1	Transcription Factor
Glucocorticoid receptor (GRE)	12.2	TS	3	Transcription Factor
Nuclear factor of activated T-cells, cytoplasmic (NF-ATc)	12.2	TS	3.1	Transcription Factor
Signal transducer and activator or transcription 3 (STAT3)	A549	TS	2	Transcription Factor
Signal transducer and activator or transcription 4 (STAT4)	A549	TS	2.3	Transcription Factor
Upstream transcription factor (USF-1)	A549	TS	2.5	Transcription Factor
Transcription co-activator Sp110	A549	SH	ND	Transcription Factor
Similar to eukaryotic translation initiation factor 2B, subunit 1 (EIF2B1)	A549	SH	ND	Translation
Insulin-like 4 (*INSL4*)	A549	SH/qPCR	27.0+/-15.8	Trophoblast [46]
Keratin 8 (*KRT8*)	A549	SH	ND	Trophoblast [47]
cDNA clone DKFZp761C1(AL157447)	A549	SH	ND	Unknown
cDNA clone FLJ20643 (AK000650)	A549	SH	ND	Unknown
FLJ14639 (NM_032815)	A549	SH	ND	Unknown
Hit clone 451B21 (AL033522)	A549	SH	ND	Unknown
HSA1p34 genomic sequence (AL009181)	A549	SH	ND	Unknown
Rhomboid family 1 (Z69719) (*RHBDF1*)	A549	SH	ND	Unknown
RP11-2H8 (AC089984)	A549	SH	ND	Unknown
RP11-389E6 (AL359836)	A549	SH	ND	Unknown
RP11-478J18 (AC011700)	A549	SH	ND	Unknown
RP11-7F24 (AC018841)	A549	SH	ND	Unknown
RP1-20C7 (AL136304)	A549	SH	ND	Unknown
SR+89 (Z69706)	A549	SH	ND	Unknown
HSA14 genomic sequence (AL135745)	12.2	SH	ND	Unknown

* Wnt/β-catenin pathway

^†^G1/S transition

Alterations in the Wnt/β-catenin pathway are commonly observed in colorectal cancers and other solid tumors, including NSCLC. We detected no differences in mRNA levels of three upstream genes in the Wnt/β-catenin pathway, disheveled (*DVL1*), adenomatosis polyposis coli (*APC*), and β-catenin (*CTNNB1*). However, transcriptional regulators downstream of β-catenin, *TCF4*, *TCF7L2*, and *LEF1*, were up-regulated in the suppressed 12.2 cell line, by 6.8-, 2.2-, and 3.4-fold, respectively (Table 3).

A549 cells are metastatic in experimental systems [24], and elevated *TSLC1 *expression is correlated with lower levels of metastasis and invasion in esophageal squamous cell carcinoma [25]. Accordingly, we examined levels of several genes involved in angiogenesis and metastasis. Increased expression of the angiogenic factor, *VEGF*, is correlated with metastasis and poor prognosis in solid tumors [26]. Surprisingly, this gene was up-regulated 4.2+/-0.3 fold in the suppressed 12.2 cells. Metalloproteinases have roles in various stages of primary tumor progression, invasion and metastasis. In A549 and 12.2, matrix metalloproteinase 1 (*MMP1*) showed no expression difference, while tissue inhibitor of metalloproteinase 1 (*TIMP1*) showed a small (2.2+/-0.2-fold) up-regulation in 12.2. These results show that some genes associated with metastasis are altered by *TSLC1*, but not necessarily as predicted by previously described pathways in tumorigenic cells.

### Proteomic Analysis of Transcription Factors

We characterized changes in transcription factor (TF) expression more broadly using the TranSignal Protein/DNA Array I (Panomics Inc., Redwood City, CA). Of the 54 TFs analyzed, 4 were down-regulated 2-fold or more in the 12.2 line and 8 were up-regulated in 12.2 (Table 3). In general, those up-regulated in 12.2 have been previously associated with repression of tumorigenesis. E2F1, which regulates the G1/S phase transition and has tumor suppressor properties, is increased in 12.2, consistent with our results from cell cycle analysis. The signal transducer and activator of transcription (Stat) proteins, which promote cellular proliferation, were down-regulated in 12.2. Interestingly, several TFs commonly disregulated in cancer, including AP-1, c-Myb, Ets, Sp1, Myc-associated factor X, NFκB and p53, were not altered between A549 and 12.2. (For complete list of TFs analyzed, see . These results are consistent with qPCR expression analysis, which did not show differences in cDNA levels for *TP53*, *MYC*, and *ETS2 *(Table 3).

### Differential gene expression analysis

Subtractive hybridization was performed between A549 cells and the suppressed 12.2 cells to identify genes differentially expressed when *TSLC1 *is restored to normal levels. The procedure was performed in both directions to produce two populations of cDNA enriched for messages up-regulated in A549 or in 12.2, respectively. Each of the differentially expressed cDNA populations was hybridized to Genome Systems cDNA arrays, identifying 41 genes greatly over-expressed in the tumorigenic A549 line and 18 genes over-expressed in the suppressed 12.2 cell line (Table 3). The differentially expressed genes represented a variety of functional classes, including those with roles in cell proliferation, cell survival, protein phosphorylation, immune response, cell adhesion, or detoxification. Several genes whose products are localized to the mitochondria were also differentially expressed.

Relative transcript levels for 31 of the differentially-expressed genes were quantified using qPCR (Table 3). Expression was normalized to glyceraldehyde 3-phosphate dehydrogenase (*GAPDH*) and alpha-tubulin (*TUBA1*). qPCR showed that *TSLC1 *expression was 2.8+/-0.8 times higher in 12.2 than in A549, as expected. Twenty-one of the 31 genes analyzed by qPCR showed expression differences of 2-fold or more in the direction predicted by subtractive hybridization. One gene, complement component C5 (*CCC5*), showed a 13.8+/-4.7-fold down-regulation in 12.2 cells, contrary to results expected after subtractive hybridization. The nine remaining genes changed less than 2-fold.

Several genes involved in cellular proliferation were among those expressed at high levels in A549 but low levels in 12.2 (Table 3). Cadherin 11 (*CDH11*), which plays a role in cell-cell interactions was also down-regulated in 12.2. Furthermore, several mitochondrial genes were down-regulated in 12.2 (Table 3). The genes that were most highly up-regulated with the restoration of *TSLC1 *expression in 12.2 cells were the candidate tumor suppressor Ras-induced senescence 1 (*RIS1*), metallothionein 1G (*MT1G*) and metallothionein 1E (*MT1E*).

### Gene expression in tumor vs. normal tissue

We compared gene expression in normal vs. tumor tissue to determine whether differences in 12.2 and A549 cells reflect changes that occur *in vivo *(Table 4). Tissue was recovered from pathological specimens and transcript levels were measured by qPCR, normalized to *GAPDH *as described [14]. *TSLC1 *and *RIS1 *levels were lower than normal in 5/5 tumor specimens, while S100 calcium-binding protein P (*S100P*) and insulin-like growth factor binding protein 1 (*IGFBP1*) levels were elevated in 5/5 and 4/5 tumors, respectively. Thus, key differences observed between the transformed (A549) and suppressed (12.2) cell lines reflect physiological differences seen in tumor vs. normal tissue.

**Table 4:** Gene expression profiles in NSCLC tumor vs. normal lung parallel those in A549 and 12.2 Relative fold expression differences, determined by qPCR, were determined in tumors and compared to normal lung tissue from same patient. Positive values represent higher expression in tumor; negative values represent higher expression in normal tissue.

	**Relative Gene Expression in Tumors**			
	
	*TSLC1*	*RIS1*	*S100P*	*IGFBP1*
	
Patient 1	-25.7	-2.3	33.3	600
Patient 2	-23.8	-6.4	2.1	2.3
Patient 3	-16.9	-7.1	28.1	-8.4
Patient 4	-344.3	-43.9	3.2	6.1
Patient 5	-32.4	-44.9	16.1	1.9
				
Cell Lines	Down in A549	Down in A549	Up in A549	Up in A549

## Discussion

Restoration of *TSLC1 *expression to normal levels in A549 cells reverses several transformed properties of this line, slowing the growth rate, restoring contact inhibition, eliminating its ability to form tumors in nude mice and blocking metastasis. In this study, we have shown that restoration of *TSLC1 *expression in 12.2 cells reduced cell growth by 3.6-fold. This change in growth rate dynamics was not due to an increase in the number of apoptotic cells. Rather, flow cytometry revealed that 12.2 cells experience a delay relative to A549 cells in progression from G1 to S phase of the cell cycle. These differences in growth related properties between A549 and 12.2 were similar to those observed previously when tumor cells were transfected with a vector containing *TSLC1 *cDNA or genomic clone [7,8,25]. These phenotypic differences led us to examine expression of genes associated with adhesion, invasion, basal metabolism, cell growth, senescence and apoptosis in A549 and 12.2 to identify classes of genes altered by restoration of *TSLC1 *expression in 12.2.

The most highly up-regulated gene in 12.2 cells was the putative tumor suppressor, Ras-induced senescence 1 (*RIS1*). *RIS1 *is located at 3p21.3, which is a region that is deleted in many human tumors [27]. Also, *RIS1 *is located within a 1 Mb human chromosomal region that is commonly deleted during tumor formation in human/mouse microcell hybrids that are passaged through severe combined immunodefficient (SCID) mice [28]. This region, called chromosome 3 common eliminated region 1 (CER1), has been posited to contain one or more currently unidentified tumor suppressors. Our results provide support for *RIS1 *as a candidate.

Coordinate up-regulation of *RIS1*, metallothionein II, and *TIMP1 *was observed in Ras-induced senescent cells [29]. Consistent with this study, we found increased levels of *RIS1*, several metallothioneins, and *TIMP1 *in 12.2 cells. Activation of these genes suggests that *TSCL1*-mediated inhibition of tumorigenesis may be related to the Ras-induced senescence pathway. However, previous studies showed that *RIS1 *expression is dependent on *ETS2*, an inducer of Ras-induced senescence, in human fibroblast IMR90 cells [29]. *ETS2 *was not differentially expressed between A549 and 12.2 cells in this study (Table 3) suggesting that *RIS1 *is activated in 12.2 cells through a different pathway.

Several genes were identified that have known roles in cancer. *S100P*, which was down-regulated in the non-tumorigenic 12.2 cell line, is involved in cell growth and has been previously implicated in prostate [30] and breast cancer progression [31]. It is over-expressed in lung adenocarcinomas [32], which frequently exhibit reduced levels of *TSLC1 *expression[7]. However, down-regulation of *S100P *in A549 using antisense RNA was not sufficient to alter growth related phenotypes by itself (data not shown). Insulin-like growth factor 4 (*INSL4*), also down-regulated in 12.2, has been reported to be over-expressed in highly invasive breast cancer cells [33]. Reduced expression of *S100P *and *INSL4 *in 12.2 may contribute to the slower growth rate and loss of tumorigenic properties in the 12.2 cell line when *TSLC1 *expression is restored to normal levels.

Several differentially expressed genes identified in this study have been previously shown, in non-overlapping experiments, to be differentially expressed during various stages of endometrial stromal cell decidualization and trophoblast implantation (Table 3). The relationship between these processes and neoplastic transformation in NSCLC is not clear. However, it is interesting that these seemingly unrelated events show similar patterns of gene expression changes. Decidualization and implantation are characterized by high levels of proliferation and tissue invasion; properties shared with transformed cells. Together, these observations suggest that *TSLC1 *may repress transformed growth via some of the same pathways that regulate proliferation in endometrial cells during various stages of decidualization.

Subtractive hybridization and qPCR showed that restoration of *TSLC1 *lowered expression of *CDH11*, an adhesion protein that may enhance cellular invasion [34]. *CDH11 *is expressed in highly invasive but not in noninvasive breast cancer cell lines [34,35]. It has been shown to associate with β-catenin (*CNNTB1*)[36]. While we did not see a significant difference in expression of upstream genes in the Wnt-1/β-catenin pathway (*DVL1, CDKN2A*, *CNNTB1*) between A549 and 12.2, we did see expression differences in *CTNNB1*-dependent transcription factors *LEF1*, *TCF4*, and *TCF7L2*, which were up-regulated in 12.2. This may be a consequence of down-regulation of *CDH11 *leading to lower levels of *CNNTB1 *sequestered at the plasma membrane. This unbound *CNNTB1 *could then translocate to the nucleus to activate downstream genes. Retera *et. al*. [37] demonstrated that *CNNTB1 *expression is reduced in NSCLC primary tumors and metastases. Our results suggest that downstream effectors of *CNNTB1*, such as *LEF1*, *TCF4*, and *TCF7L2*, may be involved in suppressing tumorigenic properties in 12.2.

Although the growth difference in A549 and 12.2 is characterized by a significant delay at G1/S in the latter, we did not find significant changes in gene expression for common G1/S phase regulators *HRAS*, *p19*, *RB1*, *TP53*, *MYC*, and *CCND1*. Also, the increased expression of *VEGF *in 12.2 cells contrasts with observations from many tissues which show that this gene is up-regulated in tumor cells. This suggests that *TSLC1 *does not suppress tumorigenesis through any of these common pathways. However, several of these genes are regulated at the protein level or through localization to the nucleus. In order to address this concern, we examined c-Myc and cyclin D1 protein levels by western blot, and found no difference in expression between A549 and 12.2 (data not shown). Furthermore, the Panomics TranSignal Protein/DNA Array found no difference in expression of Myc-associated factor X, NFκB, c-Myb, and AP-1.

Cell lines are artifactual by definition, and they do not perfectly replicate *in vivo *conditions. However, comparison of key gene expression patterns in matched tumor-normal tissue pairs showed that our results with A549 and 12.2 are representative of *in vivo *expression levels. These results validate the physiological relevance of our *in vitro *expression analysis in a model system that is far more amenable to experiment than is the minute amount of material recovered from histological specimens.

It is notable that acute expression of high levels of *TSLC1 *in A549 cells has a somewhat different effect on cell cycle profiles than does the long-term restoration of this gene in 12.2 cells. Infection for 3 or 5 days with adenovirus vectors expressing a *TSLC1 *cDNA (Ad-TSLC1) induced apoptosis and increased annexin V staining in infected cultures [38]. This contrasts with stable restoration of *TSLC1 *expression in the 12.2 line, which does not demonstrate elevation in annexin V staining. Since the 12.2 cell line was selected after transfection of *TSLC1*, it adds valuable insights into the normal function of *TSLC1 *in non-transformed cells. *TSLC1 *has the ability to suppress the transformed growth properties of A549, and it alters the gene expression profile of A549 to resemble that of normal relative to transformed lung tissue. A part of its normal function as a potent tumor suppressor may be to regulate cell growth by initiating apoptosis in those rare cells that initiate neoplastic transformation.

## Conclusion

Restoration of *TSLC1 *levels in the tumorigenic A549 cell line resulted in a loss of transformed growth properties, including a reduced cell doubling rate and a delayed progression from G1 to S phase during the cell cycle. This corresponded with a change in the gene expression profile, including changes in genes with roles in Ras-induced senescence and endometrial decidualization. Other genes with roles in cell proliferation were also altered when *TSLC1 *levels were restored, including *IGFBP1*, *S100P*, and *INSL4*. *TSLC1 *does not appear to act through any of several well-characterized cell growth regulatory pathways.

Elucidating the mechanisms by which *TSLC1 *represses tumorigenesis would have an important impact on the understanding of cancer biology in the lung, as well as in the numerous other tissues where *TSLC1 *has been associated with cancer progression. This study reveals several cellular phenotypes associated with *TSLC1 *expression and provides insights into the genes and molecular pathways induced by TSLC1.

## Methods

### Cell Culture and Tumor Samples

The A549 cell line (American Type Culture Collection [ATCC], Manassas, VA) was cultured in Dulbecco's modified Eagle's medium (Invitrogen Corp., Carlsbad, CA) supplemented with 10% fetal bovine serum (Hyclone Laboratories, Inc., Logan UT), 1X non-essential amino acids and 1% penicillin-streptomycin (Invitrogen Corp., Carlsbad, CA) in 5% CO_2_. The suppressed 12.2 cell line was created by transfecting YAC derivative y939-95 into A549 cells [8]. 12.2 cells were cultured under the same conditions as A549, with the addition of 500 μg/ml G418 (Mediatech, Inc., Herndon, VA), except for cell growth assays, in which G418 was omitted.

For the growth assays, duplicate aliquots of 5 × 10^4 ^cells were plated in six-well dishes. After 24, 48, and 120 h, cells were trypsinized and three aliquots from each well were counted using a hemacytometer. The average of six counts (three each, for two wells) is reported here.

For the WST-1 cellular proliferation assay (Roche Applied Science, Indianapolis, IN), 1 × 10^4 ^cells were cultured for 48 h. Samples were incubated with WST-1 reagent for 1 h, and absorbance was measured at 450 nm and 620 nm.

Five primary NSCLC tumors and corresponding non-cancerous lung tissues from the same patients were surgically resected and histologically diagnosed at National Cancer Center, Japan. All samples were immediately frozen after surgical resection and stored at -135°C. The analyses of human samples were carried out in accordance with the institutional guidelines.

### Proteomic Analysis

Proteomic analysis was performed using the TranSignal Protein/DNA Array I (Panomics Inc., Redwood City, CA). Nuclear protein extracts from A549 or 12.2 were incubated with an excess of biotinylated cis-binding elements (CBE) of 54 common transcription factors (TFs). Unbound CBE were removed and the protein/DNA complexes were separated, leaving labeled CBE which represent the relative protein levels of the 54 TFs. These were hybridized to a DNA array and visualized using streptavidin-HRP. Hybridized probe was quantified using the AlphaImager v5.5 software (Alpha Innotech Corp. San Leandro, CA).

### Subtractive Hybridization

Total RNA was isolated from A549 and 12.2 using Trizol reagent (Invitrogen Corp.), and poly(A) mRNA was purified using the PolyATract mRNA Isolation System II (Promega, Madison, WI). Subtractive hybridization was performed between A549 and 12.2 in both directions using the PCR-Select cDNA Subtraction Kit (Clontech, Palo Alto, CA).

The enriched pools of cDNA were hybridized to human Gene Discovery Array cDNA nylon filters (Genome Systems, Inc., St. Louis, MO). Samples (15 μl) of the final PCR reaction from each subtractive hybridization reaction were radioactively labeled by random prime-labeling with [^32^P]dCTP [39,40] and purified using ProbeQuant G-50 Sephadex columns (Amersham Pharmacia Biotech, Inc., Piscataway, NJ). The filters were prehybridized for 2 h at 42°C in buffer consisting of 0.75 M NaCl, 0.1 M Na_2_HPO_4_, 0.1% Na_4_P_2_O_7_-10H_2_O, 0.15 M Tris (pH 7.5), 5X Denhardt's solution, 2% SDS, and 100 μg/ml sheared salmon testis DNA (Sigma-Aldrich, St. Louis, MO). Probes were hybridized overnight at 42°C in the same buffer.

The membranes were washed in 2X SSC for 5 min at room temperature, twice in 2X SSC with 1% SDS for 30 min at 68°C, and twice in 0.6X SSC with 1% SDS for 30 min at 68°C. The filters were then rinsed in room-temperature 2X SSC and placed on film for 3 days for the A549 over-expressed population and 2 weeks for the 12.2 over-expressed isolates. Identities of associated EST sequences for positive clones were obtained from the Genome Systems website . EST sequences were analyzed by BLAST, using the non-redundant database to obtain gene annotation for positive clones.

### Quantitative PCR

RNA was isolated from A549, 12.2, or the antisense clones with Trizol reagent (Invitrogen Corp.) and used to generate cDNA using Superscript II reverse transcriptase (Invitrogen Corp.). Quantitative PCR (qPCR) was carried out using the LightCycler rapid thermal cycler system and the SYBR Green FastStart PCR kit (Roche Diagnostics Ltd., Lewes UK). Primers were used at 0.5 μM and MgCl_2 _at 4 mM. Samples were heat-denatured for 10 min., then cycled 55 times at 95°C for 10 sec., 58°C for 5 sec., and 72°C for 20 sec. At the completion of the cycling, a melting curve analysis was performed to detect the presence of multiple products. A standard curve was generated based on serial dilutions of PAC 66B10 and primers for marker 66B10.SP6 (CCTGGTAGTGGATTTCCCAA and ATGCCATTCAGTTTGTTCCC). Samples were normalized to glyceraldehyde 3-phosphate (ACCACAGTCCATGCCATCAC and TCCACCACCCTGTTGCTGTA). Primers for each gene were designed using the Primer3 program .

### Flow Cytometry

For the apoptosis assay, A549 and 12.2 cells were grown without antibiotic selection. Cells were trypsinized and stained with annexin V and propidium iodide as recommended (BD Biosciences Pharmingen, San Diego, CA). Cells were analyzed on a Becton Dickinson FACScan.

For cell cycle studies, 2 × 10^6 ^cells were collected and resuspended in 1 ml cold PBS, and 4 ml of -20°C 100% ethanol was slowly added. Cells were stored at -20°C overnight, recovered by centrifugation and resuspended in 1 ml PBS. RNase A (20 μg/ml) (Sigma-Aldrich Chemical Co., St. Louis, MO) was added, and the samples were incubated at 37°C for 30 min. Samples were incubated in 100 μg/ml propidium iodide (Sigma-Aldrich Chemical Co.) at room temperature for at least 1 h prior to analysis on a Becton Dickinson FACScan.

## Authors' contributions

TES carried out qPCR, protein analysis, flow cytometry, and drafted the manuscript. MTP performed the subtractive hybridization. YM acquired and analyzed tumor and normal lung tissue. RHR was responsible for the study design and coordinated data analysis.
